# Soft Substrates Promote Homogeneous Self-Renewal of Embryonic Stem Cells via Downregulating Cell-Matrix Tractions

**DOI:** 10.1371/journal.pone.0015655

**Published:** 2010-12-13

**Authors:** Farhan Chowdhury, Yanzhen Li, Yeh-Chuin Poh, Tamaki Yokohama-Tamaki, Ning Wang, Tetsuya S. Tanaka

**Affiliations:** 1 Department of Mechanical Science and Engineering, University of Illinois at Urbana-Champaign, Urbana, Illinois, United States of America; 2 Department of Animal Sciences, University of Illinois at Urbana-Champaign, Urbana, Illinois, United States of America; 3 Institute for Genomic Biology, University of Illinois at Urbana-Champaign, Urbana, Illinois, United States of America; University of Hong Kong, Hong Kong

## Abstract

Maintaining undifferentiated mouse embryonic stem cell (mESC) culture has been a major challenge as mESCs cultured in Leukemia Inhibitory Factor (LIF) conditions exhibit spontaneous differentiation, fluctuating expression of pluripotency genes, and genes of specialized cells. Here we show that, in sharp contrast to the mESCs seeded on the conventional rigid substrates, the mESCs cultured on the soft substrates that match the intrinsic stiffness of the mESCs and in the absence of exogenous LIF for 5 days, surprisingly still generated homogeneous undifferentiated colonies, maintained high levels of *Oct3/4*, *Nanog*, and Alkaline Phosphatase (AP) activities, and formed embryoid bodies and teratomas efficiently. A different line of mESCs, cultured on the soft substrates without exogenous LIF, maintained the capacity of generating homogeneous undifferentiated colonies with relatively high levels of *Oct3/4* and AP activities, up to at least 15 passages, suggesting that this soft substrate approach applies to long term culture of different mESC lines. mESC colonies on these soft substrates without LIF generated low cell-matrix tractions and low stiffness. Both tractions and stiffness of the colonies increased with substrate stiffness, accompanied by downregulation of *Oct3/4* expression. Our findings demonstrate that mESC self-renewal and pluripotency can be maintained homogeneously on soft substrates via the biophysical mechanism of facilitating generation of low cell-matrix tractions.

## Introduction

Embryonic stem cells (ESCs) are artificial stem cells that have adapted to the *in vitro* culture environment. Since the first isolation of mouse ESCs (mESCs) in 1981, mESCs have served as an excellent model to understand the mechanism of cell fate decision in developing embryos. However, the research encounters unrelenting challenges in keeping them undifferentiated homogeneously and directing their specific differentiation *in vitro*. Many studies over the years have demonstrated that undifferentiated mESC culture contains heterogeneous populations which are identified by fluctuating expression of transcripts and cell-surface markers [1]–[10]. Thus, well-accepted culture conditions are limited in maintaining self-renewal and pluripotency of mESCs [11]–[13] and human ESCs (hESCs) [14]–[16].

The importance of physical microenvironments in regulating stem cell differentiation is becoming evident nowadays [17]–[23]. Recently, we have demonstrated that mESCs are intrinsically soft and respond optimally to physical forces when cultured on substrates that match their intrinsic softness [24]. Here we demonstrate that mESCs maintain their pluripotent state optimally on the soft matrix via the mechanism of generating low cell-matrix tractions and low stiffness.

## Results

### Culturing mESCs on soft substrates generates homogeneous colonies

**Figure 1:**
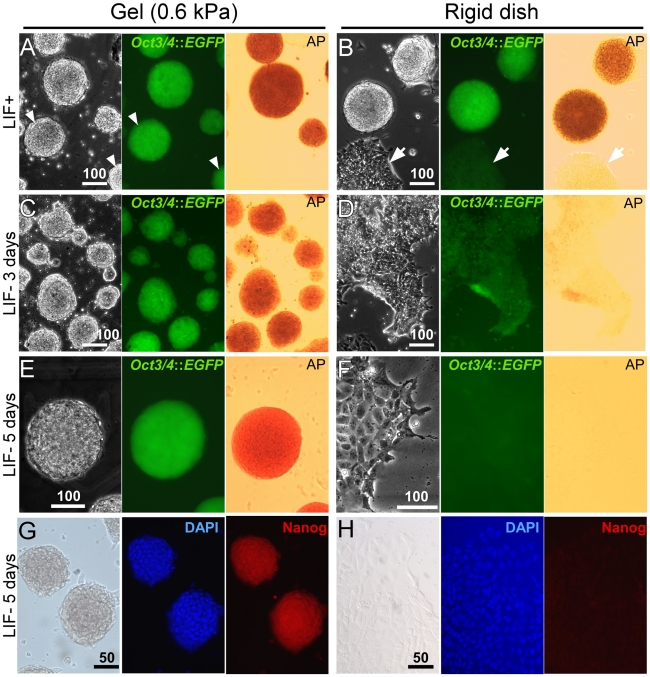
Soft substrates promote mouse embryonic stem cell (mESC) self-renewal. (A) mESCs on the substrates of 0.6 kPa stiffness [Gel (0.6 kPa)] always formed round and compact colonies (left) with uniform *Oct3/4*::GFP expression (middle) and the high AP activity (right) in the presence of LIF (LIF+). Arrowheads indicate that marked colonies were washed out during the staining procedure to measure the AP activity. (B) mESCs on the rigid substrates of polystyrene dishes (Rigid dish) with LIF formed round colonies and a spread irregular colony (left; white arrows) with heterogeneous *Oct3/4*::GFP expression (middle) and varying degrees of the AP activity (right). (C) mESCs on the soft substrates without LIF for 3 days (LIF− 3 days) still formed round colonies with uniform *Oct3/4*::GFP expression and the AP activity maintained. (D) mESCs on the rigid dish without LIF for 3 days exhibited irregular spread colonies with *Oct3/4::*GFP expression and the AP activity reduced dramatically. (E) The soft substrates supported mESC self-renewal without LIF for 5 days (LIF− 5days) with high uniform *Oct3/4*::GFP expression and the AP activity maintained. (F) On the rigid dishes, 5 days of culture without LIF resulted in irregular spread colonies with extremely low *Oct3/4*::GFP expression and a undetectable AP activity.(G–H) Immunocytochemistry with mESCs maintained on the soft (G) or the rigid substrates (H) without LIF for 5 days. Images for bright field (left) and nuclear staining with DAPI (middle) show appearance of colonies. High Nanog expression was observed in the mESCs on the soft substrates (G, right), but not in the ones on the rigid dish (H, right). Three independent experiments showed very similar results. Bars, 100 (A–F) or 50 (G & H) µms.

To explore the potential role of substrate stiffness on mESC self-renewal, we plated mESCs on soft substrates of 0.6 kPa polyacrylamide gels (referred to “gels” hereafter) that matches the intrinsic mESC stiffness or on rigid substrates of polystyrene dishes (stiffness>4 MPa) [25]; both were coated with of type-1 collagen (collagen-1), which is known to facilitate mESC self-renewal [26], under the standard culture conditions including LIF and animal serum. These mESCs express EGFP under the *Oct3/4* (*Pou5f1*) promoter (*Oct3/4*::GFP) [27]. As the mESCs were continuously cultured to form colonies, round and compact colonies were formed uniformly on the gels (pre-coated with 100 µg/ml type I collagen) with high *Oct3/4*::GFP expression and high alkaline phosphatase (AP) activities (Fig. 1A). In contrast, the mESCs plated on rigid dishes (pre-coated with 40 µg/ml type I collagen) exhibited appearances of heterogeneous colony shapes, and varying levels of *Oct3/4*::GFP expression and AP activity (Fig. 1B). Similar results were obtained when mESCs were plated on rigid dishes coated with 100 µg/ml collagen-1 (Text S1, Fig. S1), suggesting that these colonies' heterogeneous shapes and low levels of *Oct3/4* expression and AP activity are due to the rigidity of the dishes, and not due to the number of the attached collagen-1 molecules. These data were confirmed in freshly thawed mESCs: on soft substrates (Fig. S2C, D), homogenous round and compact colonies corresponded to high expressions of *Oct3/4*::GFP (Fig. S2C', D'), whereas mESCs plated directly on rigid dishes generated heterogeneous colonies of varying shapes (Fig. S2A, B), corresponding to varying expression levels of *Oct3/4*::GFP (Fig. S2A', B'). Interestingly, the mESCs plated on mouse embryonic fibroblast (MEF) feeder cells exhibited various shapes of colonies ranging from very round to somewhat flattened (Fig. S2E, F), corresponding to heterogeneous expression levels of *Oct3/4*::GFP (Fig. S2E', F'). The differences in colony shapes and *Oct3/4* expression between the mESCs on soft substrates and the mESCs on MEFs may be resulted from the fact that MEFs are much stiffer (∼10-fold) than mESCs [28]. To compare different shapes between colonies on different substrates, we measured the shape factor of mESC colonies and found that mESCs on the soft gels are much more circular than those on the rigid dishes or on the feeder cells (Fig. S2G).

**Figure 2:**
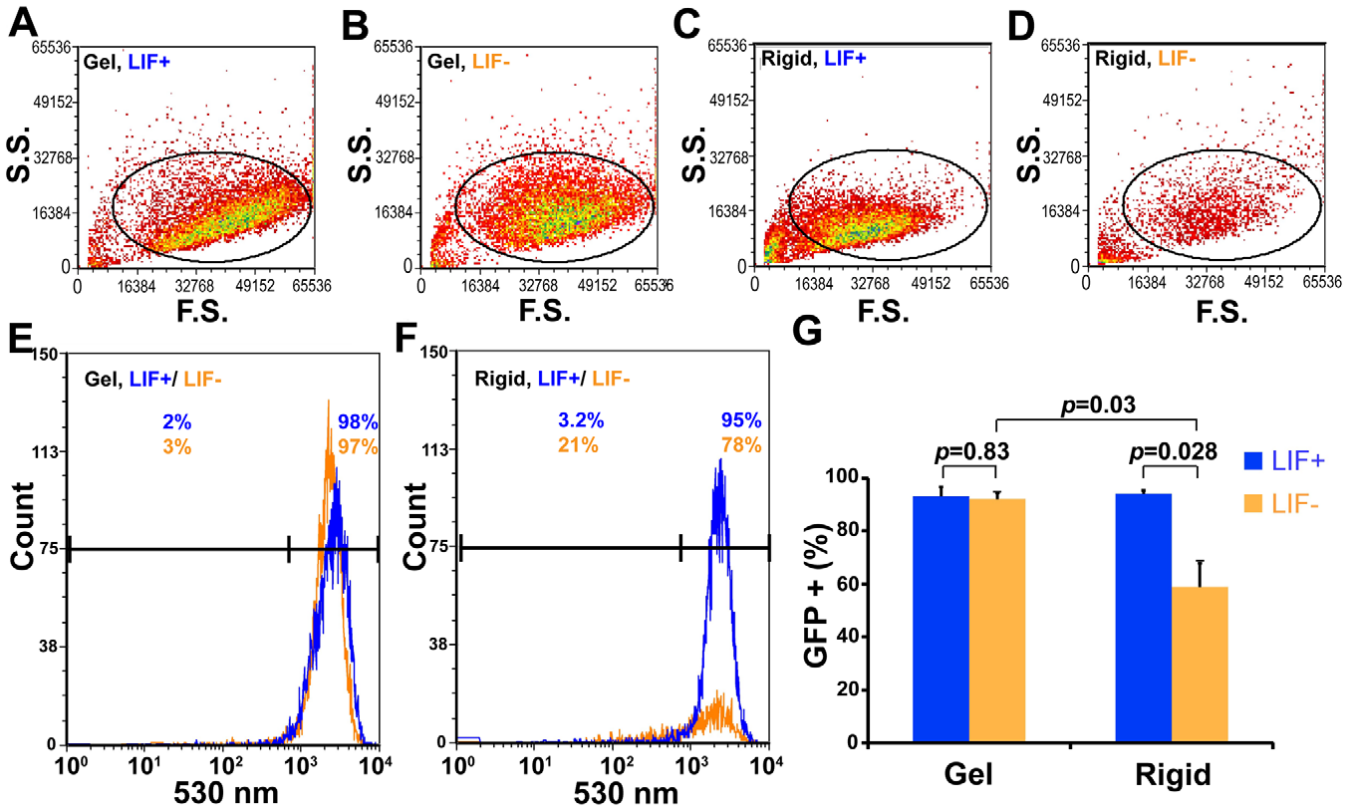
Quantification of *Oct3/4* expression mESCs on soft substrates or rigid substrates. (A–D) Representative density plots for FACS (fluorescence-activated cell sorting) of mESCs in each condition are shown. The x-axis is for forward scatter and the y-axis, side scatter. An identical gate was applied to all conditions. LIF− condition on rigid dishes yields less number of cells as some cells lose adhesion and float away [52]. This can be seen in the density plot in (D). (E) Representative plots showing high *Oct3/4*::GFP expression (530 nm) found in cells maintained in the presence (blue) or absence (orange) of LIF. The threshold of GFP expression is arbitrarily determined according to the result from sorting wild-type mESCs (W4) that do not express any fluorescent protein. Two or three percentages of sorted mESCs on the soft substrates with or without LIF are GFP-negative, respectively. (F) The percentage of GFP-negative mESCs increased to 21% of sorted mESCs on the rigid substrates without LIF from 3.2% of those with LIF. (G) Data summary shows *Oct3/4*::GFP-positive mESCs on the soft substrates or the rigid substrates with or without LIF. An identical gate was applied to all replicates. Mean ± s.e. (n = 4); at least three independent experiments.

To further explore the effect of the substrate stiffness on mESC culture, we withdrew LIF from the culture for 3 days (LIF− 3 days). Interestingly, mESCs cultured on the gels were still capable of forming round and compact colonies with the *Oct3/4*::GFP expression and the AP activity was maintained (Fig. 1C); remarkably, even in the absence of LIF for 5 days (LIF− 5 days), mESCs on gels still maintained high levels of *Oct3/4*::GFP, Nanog, and the AP activity (Fig.1E, G). In sharp contrast, the mESCs on rigid substrates in LIF− 3 days started to exhibit signs of cell differentiation with significantly reduced *Oct3/4*::GFP expression and the AP activity (Fig. 1D); as expected, in LIF− 5 days, these mESCs exhibited appearances of differentiated cells with no detectable AP activity, nor *Oct3/4* and Nanog expression (Fig. 1F, H). These data show that soft substrates can override the LIF-Stat3 signaling pathway for at least 5 days in maintaining mESC self-renewal. Next, we compared the percentage of *Oct3/4*::GFP-positive mESCs cultured on the gels with those on rigid substrates. Remarkably, almost all mESCs (92%) cultured on the soft gels maintained high *Oct3/4*::GFP expression levels in LIF− conditions (Fig. 2 A–G), similar to those in LIF+ conditions (93%) (*p* = 0.83). In contrast, when LIF was withdrawn from the culture of mESCs on rigid substrates for 5 days, *Oct3/4*::GFP-positive mESCs significantly decreased (from 94% to 59%, *p*<0.029; Fig. 2A–G). Taken together, these results indicate that the substrate stiffness is a crucial extrinsic factor to sustain the self-renewal of mESCs.

### Pluripotency of mESCs is maintained on soft substrates

**Figure 3:**
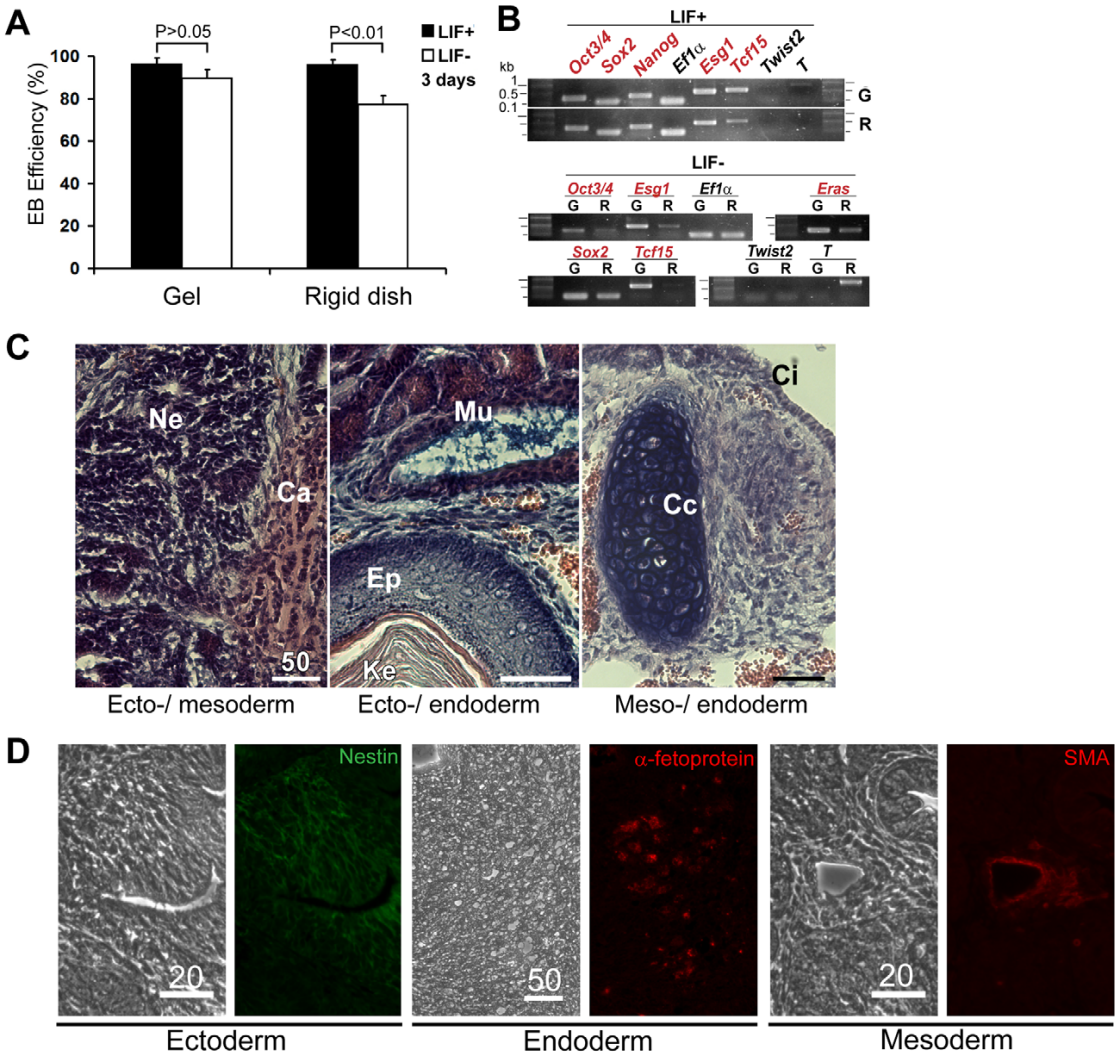
Functional validation and transcript analysis of mESCs on soft substrates. (A) Efficiencies of embryoid body (EB) formation are compared among of mESCs cultured on soft substrates and rigid dishes with or without LIF. mESCs on soft substrate retain higher EB forming capacity even in the absence of LIF as compared to those on rigid dishes. (B) Semi-quantitative RT-PCR was carried out with cDNAs from mESCs cultured in LIF+ and LIF− medium for 5 days either on the soft substrates (G) or the rigid substrates (R). Expression of pluripotency markers *Oct3/4, Esg1, Sox2 and Tcf15*, the pan-mesodermal maker *Brachyury* (*T*), the late mesodermal maker *Twist2*, and the tumorigenic marker *Eras* were analyzed. *Ef1α* is a loading control. Duplicates showed similar results. (C) mESCs cultured on soft substrates without LIF for 5 days developed a teratoma, when injected into NOD-SCID mice subcutaneously, giving rise to all three germ layers. Ne, neural tissue; Ca, cartilage; Mu, Mucous membrane; Ep, epidermis; Ke, keratin pearl; Cc, chondroitin sulfate-rich cartilage; Ci, ciliated epithelium. Bars, 50 µm. (D) mESCs cultured on soft substrates with LIF for 5 days developed a teratoma. The paraffin-embedded teratoma sections confirmed the presence of all three germ layers by immunostaining (nestin: ectoderm, α-fetoprotein: endoderm, and α-smooth muscle actin: mesoderm). Bars, 20 and 50 µm as indicated.

Because mESCs can self-renew efficiently on soft substrates, we asked whether mESCs cultured on soft substrates are still pluripotent or not. The efficiency of these mESCs to form embryoid bodies (EBs) from hanging drops was examined [29]. There were no significant differences in the efficiencies of EB formation for mESCs on soft gels with or without LIF (p>0.25); more than 90% of the hanging drops made with the mESCs formed EBs. In sharp contrast, EBs were formed in only 77% of the drops made with the mESCs maintained on rigid substrates without LIF, compared with more than 90% of the drops with the mESCs cultured on rigid substrates with LIF (*p*<0.01, Fig. 3A).

Next, we examined expression of genes associated with the undifferentiated state of mESCs (*Oct3/4*, *Sox2*, *Nanog*, *Esg1*/*Dppa5*, and *Tcf15*) as well as the genes associated with cell differentiation (*Twist2* and *T/Brachyury*) [30] in mESCs cultured on the soft gel or on the rigid substrate with or without LIF (Fig. 3B). Semi-quantitative reverse transcriptase-polymerase chain reaction (RT-PCR) data demonstrated that, in the presence of LIF (LIF+), there were no significant differences between the mESCs cultured on the gels and rigid substrates (Fig. 3B, top). However, in the absence of LIF for 5 days (LIF−), the mESCs on the soft gel still maintained high expression levels of *Oct3/4*, *Sox2*, *Esg1* and *Tcf15*, which were significantly downregulated in the mESCs on the rigid substrate (Fig. 3B, bottom). Cell differentiation was evident in the mESCs on the rigid substrate because the early mesodermal marker, *T*, was upregulated dramatically (Fig. 3B, bottom). However, *Twist2*, a late mesodermal marker, was not activated in mESCs on either the soft gels or the rigid substrates (Fig. 3B, bottom), consistent with the fact that the mESCs on the rigid substrates without LIF were in the very early stages of differentiation. Noticeably, the high expression level of the gene responsible for tumorigenic growth of mESCs, *Eras* [31], was still maintained in the mESCs on the soft gel in LIF− conditions (Fig. 3B, middle). This finding led to our investigation into the formation of teratomas by these mESCs.

When mESCs on the soft gel with LIF were transplanted to NOD-SCID mice subcutaneously for 6 weeks, they grew into a well-developed teratoma (Fig. S3A, dashed-circles) with cell types of three germ layers (Fig. S3C-E). As expected, teratomas were formed when the mESCs on the rigid substrate with LIF were transplanted. Intriguingly, when the mESCs maintained on the gels without LIF for 5 days were transplanted for 7 weeks, they were able to grow into a well-developed teratoma (Fig. S3B, dashed-circle on the left) consisting of cell types of three germ layers, much larger than the teratoma generated from the mESCs on the rigid substrate without LIF (Fig. S3B, dashed-circle on the right). This result is consistent with the high expression level of *Eras* in the mESCs on the soft gel without LIF and the low expression level of *Eras* in the mESCs on the rigid substrate without LIF (Fig. 3B).

To determine if our approach could be extended to other mESC lines and for long term cultures, we initiated culture of another established line of mESCs (W4, 129/SvEv). Remarkably, after W4 mESCs were passaged more than 15 times on the soft gels without exogenous LIF continuously for more than 2 months, they still exhibited round, compact colonies with relatively high levels of *Oct3/4* expression and the AP activity (Fig. S4, row 3). In contrast, W4 mESCs cultured on the rigid dishes for the same duration, even in the presence of LIF, exhibit irregular shapes of colonies with some differentiated cells at the periphery of the colony and with low levels of AP activity and *Oct3/4* expression (Fig. S4, row 1). These results demonstrate that the soft substrate strategy to promote self-renewal of ESCs could be applied to other mESCs for long term cell cultures.

### A biophysical mechanism of substrate softness mediated mESC self-renewal

**Figure 4:**
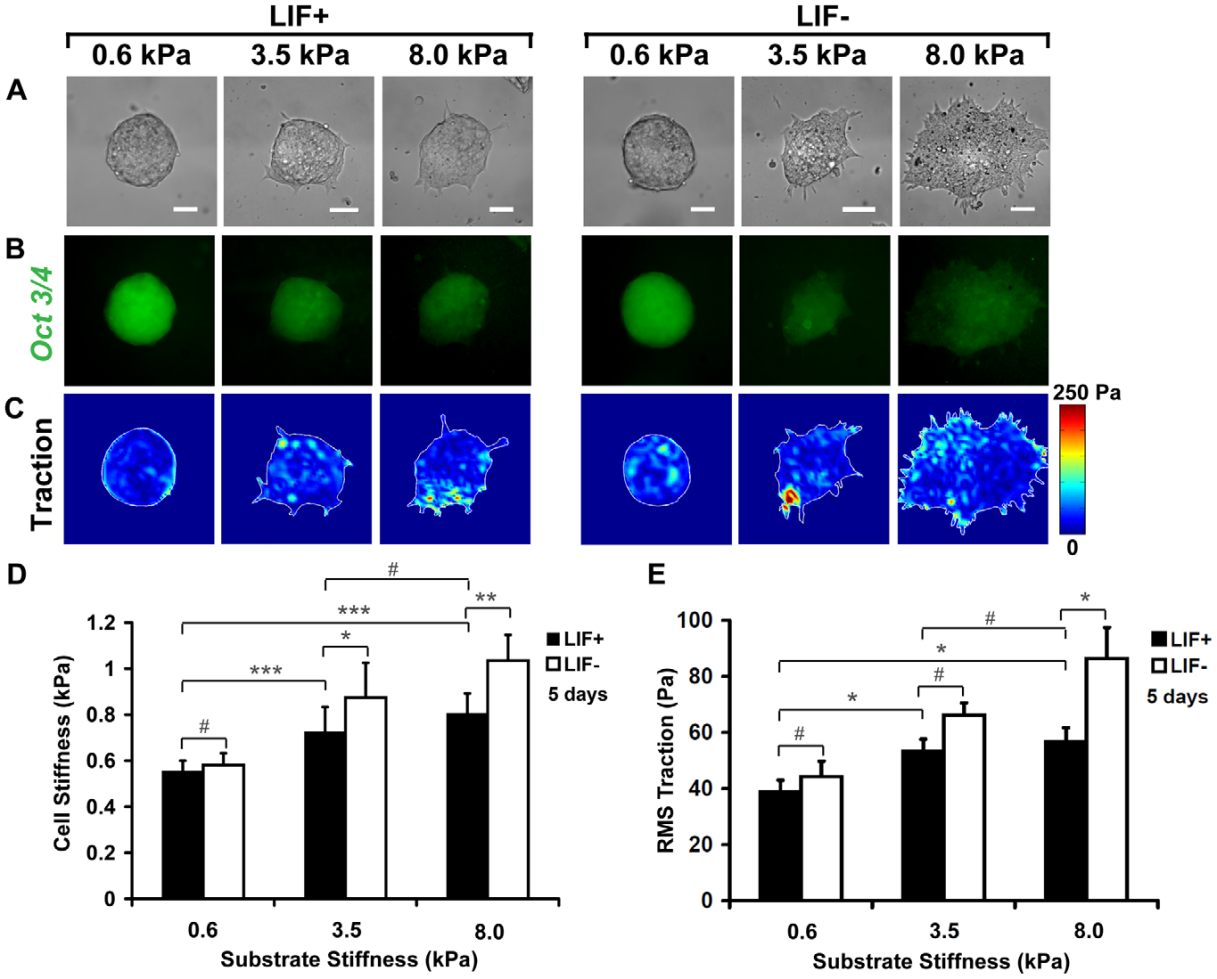
Elevated endogenous stress and stiffness lead to mESC differentiation. (A) Bright-field images of colonies on 0.6, 3.5 or 8 kPa substrates with or without LIF. Colonies are round and compact on 0.6 kPa substrates in the presence and absence of LIF. In contrast, colonies on 3.5 kPa, similar to 8 kPa substrates, are spread in the presence of LIF and even more spread and irregular in the absence of LIF. (B) Corresponding GFP images of *Oct3/4* expression of the same colonies on 0.6, 3.5 or 8 kPa substrates. Uniform *Oct3/4*::GFP expression is found in colonies on 0.6 kPa substrates but not on 3.5 and 8 kPa substrates. (C) Colonies on 0.6 kPa substrates exert lower tractions than colonies on 3.5 and 8 kPa substrates. (D) Summarized data shows that stiffnesses of the colonies are significantly different between 0.6 and 3.5 kPa substrates, and between 0.6 and 8 kPa substrates, but similar between 3.5 and 8 kPa (all are in LIF+ conditions). Colony stiffnesses are similar with (n = 52) or without (n = 50) LIF on 0.6 kPa substrates, but are significantly different between with (n = 22) or without (n = 19) LIF on 3.5 kPa, and on 8 kPa substrates (n = 85, 10 colonies with or without LIF). Mean ± s.e. (E) RMS (root-mean-square) tractions of colonies on 0.6, 3.5 or 8 kPa substrates. In the presence of LIF, when substrate stiffness increased from 0.6 kPa to 3.5 kPa or to 8 kPa, tractions significantly increased. Tractions on 0.6 kPa were similar with (n = 8) or without (n = 7) LIF; tractions on 3.5 kPa were also similar with (n = 7) or without (n = 6) LIF, but tractions on 8 kPa substrates were different with (n = 6) or without (n = 7) LIF. Mean ± s.e. Bars, 50 µm. (*, p<0.05; **, p<0.01; ***, p<0.001; #, p>0.05)

Increasing evidence suggests that matrix substrate rigidity influences cell functions via a biophysical mechanism [18], [19]. To explore the biophysical mechanism of mESC self-renewal on soft substrates, we plated mESCs on 0.6 kPa (soft), 3.5 kPa (relatively stiff), or 8 kPa (stiff; ∼10-fold greater than the intrinsic mESC stiffness) substrates in the presence or absence of LIF and allowed individual cells to grow into colonies. As observed earlier, mESCs on 0.6 kPa substrates formed round compact colonies (Fig. 4A), maintained high *Oct3/4*::GFP, with or without LIF (Fig. 4B). Tractions on the basal surface and stiffness on the apical surface of the colony did not change with or without LIF on the 0.6 kPa soft substrate (Fig. 4C–E). However, as the substrate stiffness increased from 0.6 to 3.5, and then to 8 kPa, the mESC colonies with LIF became irregular and expressed low levels of *Oct3/4* (Fig. 4A, 4B). The shapes of the colonies on the 8 kPa substrate are similar to those from the mESC colonies on the rigid substrate of polystyrene dishes, suggesting that the 8 kPa substrate and the rigid substrate are “equivalent” in rigidity in regards to mESC stiffness: stiffnesses of both substrates are much higher than mESC stiffness. The mESC colonies on 3.5 kPa substrates with LIF generated higher tractions and higher stiffness than on 0.6 kPa substrates with LIF, but similar tractions as those on 8 kPa substrates (Fig. 4C–E). This result suggests that mESCs have started to respond mechanically (changes in traction and stiffness) and biologically (changes in *Oct3/4* expression) when the substrate stiffness is increased by as little as a factor of 6 (from 0.6 to 3.5 kPa). In LIF− conditions for 5 days, the mESC colonies on 8 kPa substrates, similar to those on 3.5 kPa substrates, became much more spread and irregular, showing signs of differentiation (Fig. 4A), and significantly elevated their tractions and stiffness (Fig. 4C–E), accompanied by diminishing *Oct3/4* expression (Fig. 4B). To further examine the role of myosin II in traction generation of the colonies, we cultured mESCs on 8 kPa substrates with blebbistatin (10 µM) for 5 days. After treatment with blebbistatin to inhibit myosin II, the colonies became much more uncompact and irregular (Fig. S5A, B), and tractions were downregulated (Fig. S5C, D). Addition of blebbistatin significantly lowered the levels of *Oct3/4* expression in the colonies without LIF from the control (untreated cells with LIF). These data are consistent with a recent report that blebbistatin treatment decreases compactness and slightly downregulates *Oct3/4* expression of human ESC (hESC) colonies [32]. Together with the published reports that mouse embryos cease to develop when myosin-IIs are genetically knocked out [33], [34], differentiation of mesenchymal stem cells directed by matrix substrates is blocked when myosin-II-dependent tractions are inhibited [17], and external stress-induced mESC spreading and differentiation are inhibited by myosin-II inhibitor blebbistatin [24], our present data demonstrate that mESC colonies on soft substrates maintain their self-renewal and pluripotency via the biophysical mechanism of generating low cell-matrix tractions and low stiffness.

## Discussion

Our data show that when the stiffness of matrix substrates matches that of the soft mESCs, the soft substrate promotes self-renewal and pluripotency of mESCs, even in the absence of LIF for at least 5 days. These results demonstrate that the substrate softness plays a crucial role in the maintenance of mESC self-renewal and pluripotency. It is clear from our data that our approach can generate homogeneous mESC culture, a major advantage over the standard culture approach. Importantly, plating mESCs on soft substrates is able to override the differentiation propensity triggered by LIF withdrawal from the medium. Our discoveries on the importance of matching the material properties of the substrate with those of the mESCs on the optimal mESC self-renewal and pluripotency functions extend the previous findings in skeletal and cardiac muscle cells [35], [36] and the finding from a very recent report on skeletal muscle stem cells [37]. The generation of a homogeneous undifferentiated population of all mESC colonies on the soft substrates indicates that the current protocols to culture mESCs can be substantially improved by plating the mESCs on soft substrates. Furthermore, our data raise a potential significant impact of substrate stiffness on tumorigenesis by ESCs (Fig. S3). Understanding this role may dramatically improve the safety issue of ESCs and induced pluripotent stem cells (iPSCs) in regenerative medicine.

Recently we have shown that mESCs downregulate expression of the pluripotency marker *Oct3/4* and differentiate as increased stresses via integrins are applied externally [24]. We have recently shown that single mESCs generate low basal tractions on soft substrates and increase their basal tractions as substrate stiffness increases [38]. However, stiffness at the apical surface of single mESC does not vary with basal substrate stiffness [38]. In contrast, in this study, we show that both apical stiffness and basal tractions of mESC colonies increase with substrate stiffness, possibly due to the fact that mechanosensing capacities of the E-cadherins [39] at lateral adherens junctions have promoted mechanical interactions between the apical cytoskeleton and the basal cytoskeleton. E-cadherins have been implicated in self-renewal and pluripotency of ESCs [40], [41]. E-cadherin knockout mESCs have shown evidence of LIF independence [42]. Recently it is demonstrated that E-cadherin and myosin IIA play important roles in facilitating hESC self-renewal and survival [32]. It is possible that cell-matrix tractions and cell-cell tractions exert opposing effects on self-renewal and differentiation: high cell-matrix tractions promote differentiation whereas high cell-cell tractions promote self-renewal and pluripotency. Blebbistatin or myosin-II knockdown inhibits both cell-matrix tractions and cell-cell tractions [32]; thus the effects of these interventions on ESC pluripotency and differentiation could be complicated. We have noticed that on the same ∼8 kPa substrate, hESC colonies generate ∼10-fold higher cell-ECM tractions (RMS traction ∼600 Pa, peak traction ∼2000 Pa) [32] than mESC colonies (RMS traction ∼60 Pa, peak traction ∼200 Pa; our present study), suggesting that hESC colonies may either generate much greater total force or transfer more myosin II-dependent contractility to the matrix substrate and less force between cell-cell adhesions than mESC colonies. In the future the relationship between cell-cell adhesion E-cadherins and cell-matrix adhesion in mechanics, biology, self-renewal, and pluripotency of mESCs and hESCs needs to be elucidated. The present data also show that expression of *Oct3/4* is inversely associated with the traction and the stiffness of the mESC colonies. These findings lead us to the following question, what is the underlying mechanism by which soft-substrates can maintain self-renewal and pluripotency of mESCs? Our data demonstrate that mESC colonies maintain their self-renewal and pluripotency when the tractions and stiffness of the colonies are kept low on the soft substrate. In addition, pluripotency marker *Oct3/4* is inversely associated with the traction and the stiffness of the mESC colonies. These data indicate that mESC colonies tend to differentiate when both myosin-II dependent basal tractions and apical cell stiffness increase as the substrate stiffness increases. The findings of low tractions (prestress) in mESCs in the present study have been predicted from our previous analyses of molecular basis of mESC rheology using the model of molecular dynamics simulation and living cell rheological measurements [28]. Currently the exact underlying mechanism that connects the low traction and low stiffness on soft substrates with the self-renewal and pluripotency of mESCs is not clear. However, it is possible that genes essential to sustain cellular pluripotency are kept “turned-on” by low mechanical stresses. Once the high endogenous mechanical stresses generated on the rigid substrates are applied to the cytoskeleton and the nucleus, genes associated with cell differentiation and/or the transcription factors that regulate expression of such genes are directly activated whereas pluripotency genes are inhibited [43], [44], via the molecular mechanisms of conformational change or unfolding of cytoskeletal proteins and/or nuclear proteins [44]–[47]. This interpretation is consistent with a report that simulated microgravity promotes formation of ball-like ES cell colonies in the absence of LIF [48]. Alternatively, soft substrates may promote production of and/or cellular accessibility to LIF and/or other soluble growth factors to sustain self-renewal and pluripotency of mESCs. However, this alternative interpretation is not able to explain the fact that saturating amounts of LIF or other soluble growth factors alone fail to maintain homogenous populations of mESC colonies on rigid substrates, whereas soft substrates can. It is interesting that ROCK inhibitors that inhibit Rho-mediated cytoskeletal tension can promote self-renewal and pluripotency and reduce apoptosis of hESCs [49], consistent with our ideas on the role of low tractions on self-renewal and pluripotency of mESCs.

Collectively, we conclude that soft substrates promote self-renewal and pluripotency of mESCs primarily via the biophysical mechanism of low-traction/low-stiffness-dependent gene regulation. It remains to be seen if our findings and the underlying biophysical mechanism on mESCs can be extended to hESCs and iPSCs since recent advances in defining culture conditions chemically are not sufficient to prevent spontaneous differentiation of hESCs [50]. Several recent papers have reported the improved long term self-renewal of hESCs using synthetic surface molecules or recombinant matrix molecules [14]–[16]. However, significant challenges remains for hESC culture, since long term culture and passages of hESCs lead to significant changes of copy number variations (CNVs) and gene expressions [51]. It is conceivable that if the substrate softness would match that of the hESCs, homogenous populations of self-renewal, pluripotent hESCs might be generated for long-term without inducing changes in CNVs and/or gene expressions.

## Materials and Methods

### Cell culture

A mouse embryonic stem cell (mESC) line, namely OGR1, that expresses EGFP under the promoter of *Oct3/4* (*Oct3/4*::GFP) [27] was used in this study. These undifferentiated mESCs were maintained in the standard culture condition as described before [24] in the presence of Leukaemia Inhibitory Factor (LIF; Chemicon). Briefly, undifferentiated mESCs were cultured in the ES cell medium consisting of high glucose-Dulbecco's modified Eagles medium (Invitrogen) supplemented with 15% ES-qualified fetal bovine serum (FBS; Invitrogen), 2 mM L-glutamine (Invitrogen), 1 mM sodium pyruvate, 0.1 mM nonessential amino acids (Invitrogen), penicillin/streptomycin, 0.1 mM beta-mercaptoethanol (Sigma), and 1000 U/ml recombinant LIF (ESGRO®; Millipore) at 37°C with 5% CO2. Cells were passaged every 2–3 days at a ratio of 1:6 using TrypLE™ (Invitrogen). The medium was changed daily. For experiments, cells were plated on type I collagen (Sigma)-coated (40 or 100 µg/ml) rigid dishes or type I collagen-coated (100 µg/ml) polyacrylamide gels (0.6, 3.5, and 8 kPa) and cultured up to 5 days (unless stated otherwise) with or without LIF. The polymer layer formed by the collagen-1 molecules are too thin (<<0.2 µm) to affect the modulus of the polyacrylamide gel (∼70 µm in thickness) that an attached cell feels. For some experiments on 8 kPa substrates we added 10 µM Blebbistatin for 5 days. Blebbistatin containing medium was changed every two days as it is stable for up to 48 hours [17].

### Flow Cytometric Sorting

OGR1 mESCs were sorted on the i-Cyt Reflection system with a nozzle of 100 µm and at a rate of 3000 to 5000 cells/second at 20 psi. Under the identical culture conditions, wild-type mESCs (W4, 129S6/SvEvTac) having no fluorescent protein expression were served as a negative control. Trypsinized cells were suspended in ice-cold PBS containing 10% FBS just before each experiment.

### EB formation assay

Hanging drop cultures were prepared using 25 µl droplets each having 600 cells to initiate embryoid body (EB) formation [29]. After maintained in the presence or the absence of LIF for 3 days, mESCs were allowed to aggregate and form EBs in the bottom of the hanging drops made with the ES medium without LIF for 3 days. Then, they were transferred to adherent culture dishes. The number of EBs formed was counted and therefore the efficiency of EB formation was calculated for each test condition.

### Teratoma formation assay

One million viable mESCs (OGR1) in ice-cold 25 µl PBS together with 25 µl of 0.3 µg/ml type-I collagen were injected into NOD-SCID mice subcutaneously. Health of mice was monitored regularly. They were humanely sacrificed after 6–7 weeks (according to the protocol approved by IACUC, University of Illinois) and teratomas were isolated. These teratomas were fixed with 4% paraformaldehyde in PBS at 4°C overnight and further processed for standard Alcian Blue, Hematoxylin and Eosin (H&E) staining.

### Traction measurements

Cell traction measurements have been described in details elsewhere [53]. Briefly, images of red fluorescent submicrobeads (0.2 µm) embedded into the apical surface of gels (∼70 µm in thickness) were taken during experiments and compared with a reference image at the end of experiment after trypsinizing colonies from the substrates. The displacements of the beads were computed to generate a displacement field of the colony generating forces on the underlying substrates. A traction field was then calculated from the displacement field by an established method [54].

### Quantification of cell stiffness

Complex stiffness was measured by applying an oscillatory magnetic field (i.e., applied specific torque, T, or the applied stress = 17.2 Pa at 0.3 Hz) and measuring the resultant oscillatory bead motions (i.e., the measured strain) [24], [28], [55]–[57]. The stiffness has the units of torque per unit bead volume per unit bead displacement (Pa/nm), which is independent of any model. The beads were coated with saturating amounts of RGD (Arg-Gly-Asp) to bind specifically to integrin receptors. The beads were embedded ∼50% into the cell apical surface as shown earlier [24]. We used the 50% bead-cell surface contact area and an established finite element model to convert stiffness (Pa/nm) to modulus (Pa) [58] and determined that 1 Pa/nm stiffness is equivalent to 2.5 kPa modulus. In the analysis, only those beads whose displacement waves conformed to the input sinusoidal signals at the same frequency were selected which is essential to filter out the noise (e.g., spontaneous bead movements or microscope stage shifts). Beads with displacements less than 5 nm (limitation of resolution detection) and loosely bound beads were not selected for analysis. To increase signal to noise ratio, peak amplitude of the displacement “d” (nm) was averaged over 5 consecutive cycles.

### Gene Expression Analysis

The same amount of total RNA (1.6 µg) from mESCs in each condition was used to synthesize the first strand cDNA as previously described [59]. PCR mixtures by Phusion DNA polymerase (NEB) were prepared according to the manufacturer's instructions. The PCR conditions were as follows: first, denaturing at 98°C for 1 min, different number of cycles of denaturing at 98°C for 10 sec, annealing at 65°C for 30 sec, and extension at 72°C for 30 sec, followed by a final extension reaction at 72°C for 7.5 min. As to the PCR cycles for the samples for LIF− conditions, 16 cycles were applied for *Oct3/4*, *Esg1* and *Ef1α*; 25 cycles for *Sox2* and *Tcf15*; 27 cycles for *Twist2* and *T(Brachyury)*; 29 cycles for *Eras*.

### Polyacrylamide Substrates

Polyacrylamide substrates were made of as described before [60]. The elastic Young's modulus of the polyacrylamide substrates used in this study was 0.6 kPa (0.06% bis-acrylamide, 3% polyacrylamide), 3.5 kPa (0.1% bis-acrylamide, 5% polyacrylamide), and 8 kPa (0.3% bis-acrylamide, 5% polyacrylamide) [61], [62]. Red fluorescent microspheres (0.2 µm; Molecular Probe) were embedded onto the gels for traction measurements so that EGFP expression in OGR1 mESC colonies did not interfere with traction measurements.

### Statistical Analysis

Student's t-test was applied to all statistical analyses.

## Supporting Information

Figure S1 Mouse ESCs were plated on collagen-1 (100 µg/ml) coated rigid dishes and cultured for 5 days in LIF+/− conditions. The colonies were immunostained for Oct3/4 and the alkaline phosphatase (AP) activity. Colonies exhibited similar phenotypes to the ones maintained on 40 µg/ml collagen-1.
(TIF)

Figure S2 Mouse embryonic stem cells (mESCs; OGR1) thawed and maintained on soft gels formed round and compact colonies as they did on feeders. Bright (*A*–*F*) and dark (*A*'–*F*') field images are shown. (*A*–*A*') ORG1 mESCs thawed on rigid dishes formed small spread colonies on day 3. However, *Oct3/4*::GFP expression at this stage were not significantly diminished. (*B*–*B*') mESCs thawed on rigid dishes on day 6 showed appearance of spread and differentiated cells. The corresponding dark field image showed very low GFP expression. (*C*–*C*') mESCs thawed on the soft gels started to form round and compact colony on day 3 with GFP expression. (*D*–*D*') on day 6, these mESCs on the soft gel still formed very round and compact colonies with GFP uniformly expressed. (*E*–*E*') On day 3, mESCs thawed on feeders appeared to have colonies of various shapes ranging from relatively round to somewhat flattened (white arrow in *E*). The flattened colony showed low GFP expression (arrow in *E*'). (*F*–*F*') On day 6, mESCs formed relatively round colonies on feeders with GFP expression, except for the cells on the edge of the colony whose GFP expression was relatively low, showing early signs of differentiation. (*G*) Comparisons among the shapes of colonies on the rigid dish, the gel and the feeders by quantifying the colony shape factor [11]. The colony shape factor ( = 4πArea/Perimeter^2^; Area = colony projected area; Perimeter = perimeter length of a colony) measures to what extent the colony is similar to a true circle. A true circle has a value of unity. Data are mean ± s.e.m., *n* = 29, 32, 30 colonies for the rigid dish, the gel and the feeders respectively. *p*<0.0001 between any two conditions. Bars, 100 µm.
(TIF)

Figure S3 Mouse ESCs maintained on soft gels under LIF+ and LIF− conditions formed a well-developed teratoma when transplanted into NOD-SCID mice subcutaneously. (*A*) Teratomas (dashed circles) are developed from mESCs cultured on the soft gel in the presence of LIF. (*B*) The teratoma on *left* is developed from mESCs on the soft gel, whereas the teratoma on *right* is from ones on rigid dishes in the absence of LIF. n = 2 separate mice. The teratoma on right is significantly smaller in size. (*C*–*E*) Hematoxylin and Eosin (H & E) staining of sections from a teratoma of mESCs maintained on the soft gel with LIF shows the presence of cells from all three germ layers. Ne: Neural tissue (ectoderm); St: Striated muscle (mesoderm); Ci: Ciliated epithelium (endoderm).
(TIF)

Figure S4 Undifferentiated mouse ES cell line, W4 (129/SvEv), was serially passaged (images shown at passage 15) on rigid dishes and soft gels (0.6 kPa) under LIF +/− conditions for over three months. Even in the presence of LIF on rigid dishes, cells start to exhibit decreased *Oct3/4* expression and the AP activity accompanied by appearance of differentiated cells at the colony periphery (row 1). However, their self-renewal was maintained best on soft gels in the presence of LIF, evident by the high *Oct3/4* expression level, the high AP activity, and compact and round morphology (row 2). Remarkably, cells on soft gels also maintained self-renewal in the absence of LIF with sustained *Oct3/4* expression and the AP activity (row 3).
(TIF)

Figure S5 Blebbistatin (10 µM) treatment on 8 kPa substrates for 5 days decreases RMS tractions. (A–C) Blebbistatin treatment (Bb+) altered colony shape (A), Oct3/4 expression (B), and tractions (C). (D) For LIF+ conditions, adding blebbistatin downregulated tractions (*p* = 0.032; n = 10 colonies). Similary, for LIF− conditions, addition of blebbistatin decreased tractions (*p* = 0.03; n = 8 colonies). For convenience of comparison, data (without blebbistatin) are replots from part of Fig. 4E. Mean ± s.e.m. Bars, 50 µm. (E) Summarized data for *Oct3/4* expression after blebbistatin treatment. Control: colonies on 8 kPa with LIF (n = 9). Blebbistatin significantly lowered the level of *Oct3/4* expression in colonies without LIF (n = 6) when compared with the control (p<0.01). LIF withdrawal alone (n = 7) or blebbistatin added to LIF+ condition (n = 8) decreased *Oct3/4* expression from the control only slightly but not significantly (p>0.25). Mean+/−s.e.m.
(TIF)

Text S1 Additional Methods.
(DOC)
