# Supplementary figures and images for: Soft Substrates Promote Homogeneous Self-Renewal of Embryonic Stem Cells via Downregulating Cell-Matrix Tractions
Source: PLoS One. 2010 Dec 13;5(12):e15655. doi: 10.1371/journal.pone.0015655 (PMC3001487; doi:10.1371/journal.pone.0015655)

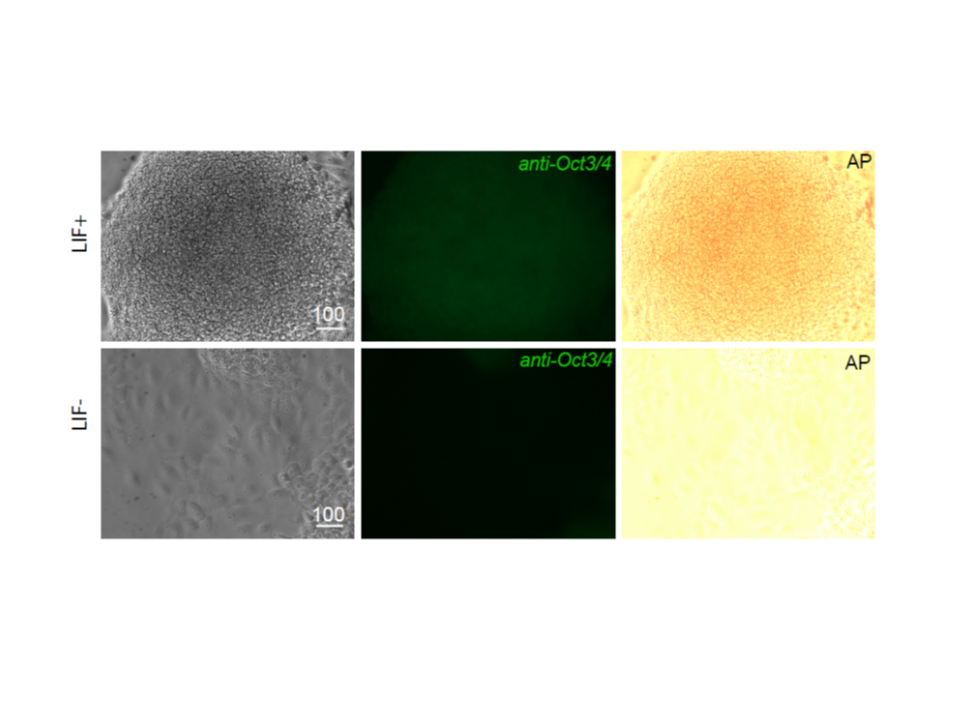

Supplement: Figure S1 — Mouse ESCs were plated on collagen-1 (100 µg/ml) coated rigid dishes and cultured for 5 days in LIF+/− conditions. The colonies were immunostained for Oct3/4 and the alkaline phosphatase (AP) activity. Colonies exhibited similar phenotypes to the ones maintained on 40 µg/ml collagen-1. (TIF) [file pone.0015655.s001.tif]

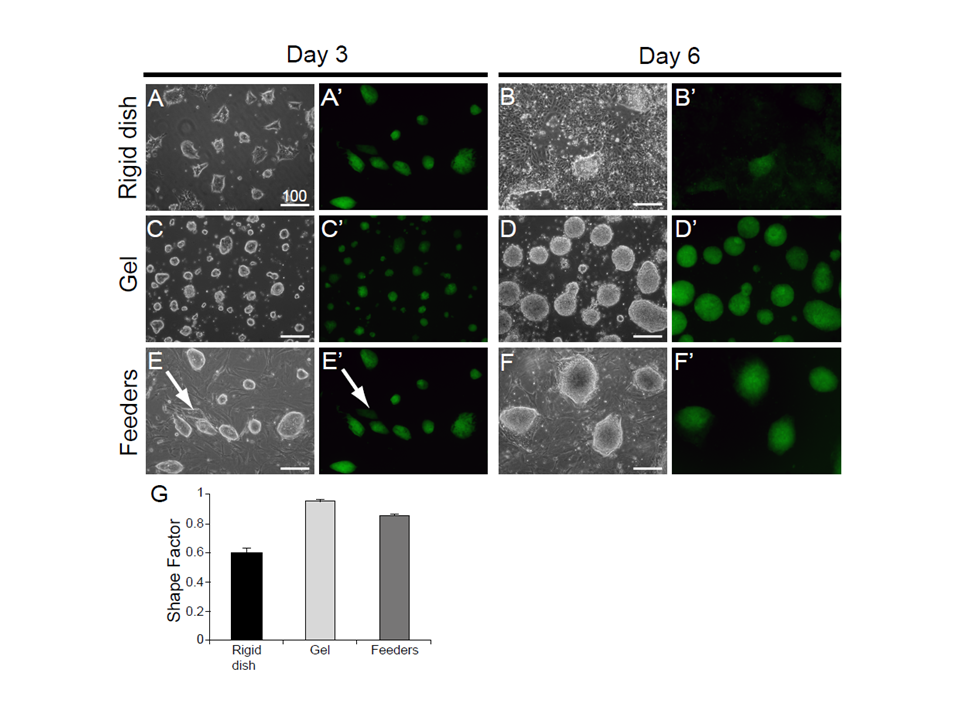

Supplement: Figure S2 — Mouse embryonic stem cells (mESCs; OGR1) thawed and maintained on soft gels formed round and compact colonies as they did on feeders. Bright (A–F) and dark (A'–F') field images are shown. (A–A') ORG1 mESCs thawed on rigid dishes formed small spread colonies on day 3. However, Oct3/4::GFP expression at this stage were not significantly diminished. (B–B') mESCs thawed on rigid dishes on day 6 showed appearance of spread and differentiated cells. The corresponding dark field image showed very low GFP expression. (C–C') mESCs thawed on the soft gels started to form round and compact colony on day 3 with GFP expression. (D–D') on day 6, these mESCs on the soft gel still formed very round and compact colonies with GFP uniformly expressed. (E–E') On day 3, mESCs thawed on feeders appeared to have colonies of various shapes ranging from relatively round to somewhat flattened (white arrow in E). The flattened colony showed low GFP expression (arrow in E'). (F–F') On day 6, mESCs formed relatively round colonies on feeders with GFP expression, except for the cells on the edge of the colony whose GFP expression was relatively low, showing early signs of differentiation. (G) Comparisons among the shapes of colonies on the rigid dish, the gel and the feeders by quantifying the colony shape factor [11]. The colony shape factor ( = 4πArea/Perimeter2; Area = colony projected area; Perimeter = perimeter length of a colony) measures to what extent the colony is similar to a true circle. A true circle has a value of unity. Data are mean ± s.e.m., n = 29, 32, 30 colonies for the rigid dish, the gel and the feeders respectively. p<0.0001 between any two conditions. Bars, 100 µm. (TIF) [file pone.0015655.s002.tif]

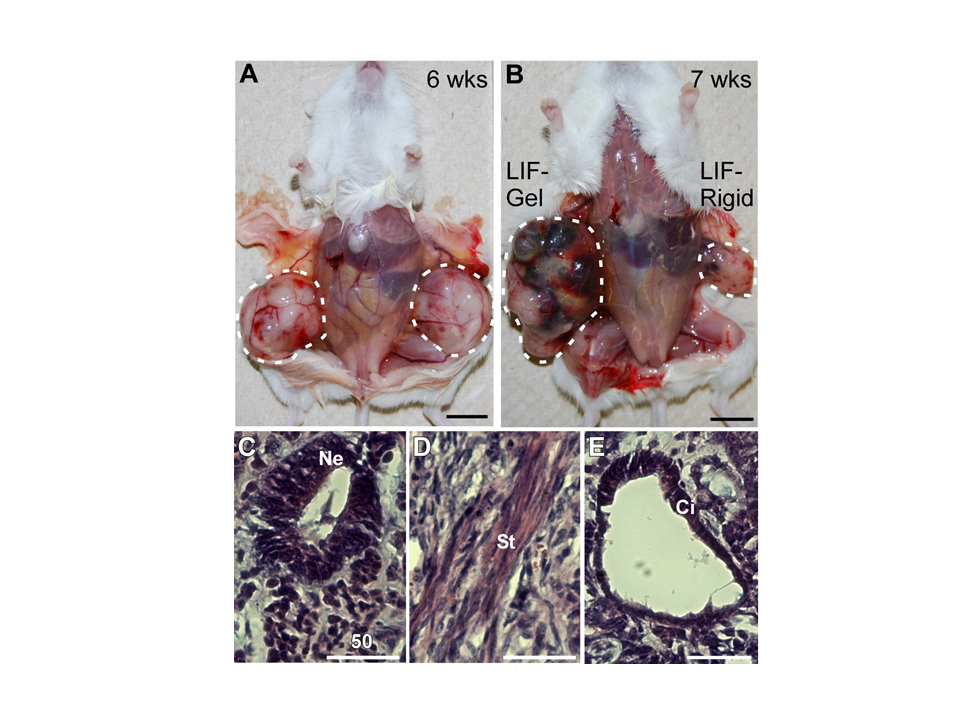

Supplement: Figure S3 — Mouse ESCs maintained on soft gels under LIF+ and LIF− conditions formed a well-developed teratoma when transplanted into NOD-SCID mice subcutaneously. (A) Teratomas (dashed circles) are developed from mESCs cultured on the soft gel in the presence of LIF. (B) The teratoma on left is developed from mESCs on the soft gel, whereas the teratoma on right is from ones on rigid dishes in the absence of LIF. n = 2 separate mice. The teratoma on right is significantly smaller in size. (C–E) Hematoxylin and Eosin (H & E) staining of sections from a teratoma of mESCs maintained on the soft gel with LIF shows the presence of cells from all three germ layers. Ne: Neural tissue (ectoderm); St: Striated muscle (mesoderm); Ci: Ciliated epithelium (endoderm). (TIF) [file pone.0015655.s003.tif]

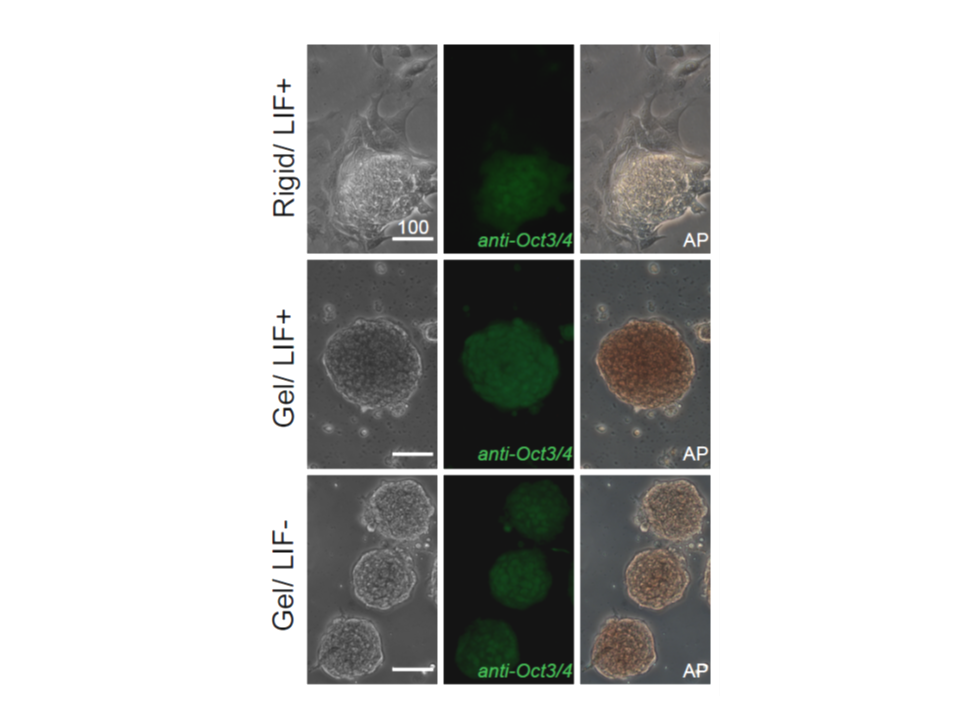

Supplement: Figure S4 — Undifferentiated mouse ES cell line, W4 (129/SvEv), was serially passaged (images shown at passage 15) on rigid dishes and soft gels (0.6 kPa) under LIF +/− conditions for over three months. Even in the presence of LIF on rigid dishes, cells start to exhibit decreased Oct3/4 expression and the AP activity accompanied by appearance of differentiated cells at the colony periphery (row 1). However, their self-renewal was maintained best on soft gels in the presence of LIF, evident by the high Oct3/4 expression level, the high AP activity, and compact and round morphology (row 2). Remarkably, cells on soft gels also maintained self-renewal in the absence of LIF with sustained Oct3/4 expression and the AP activity (row 3). (TIF) [file pone.0015655.s004.tif]

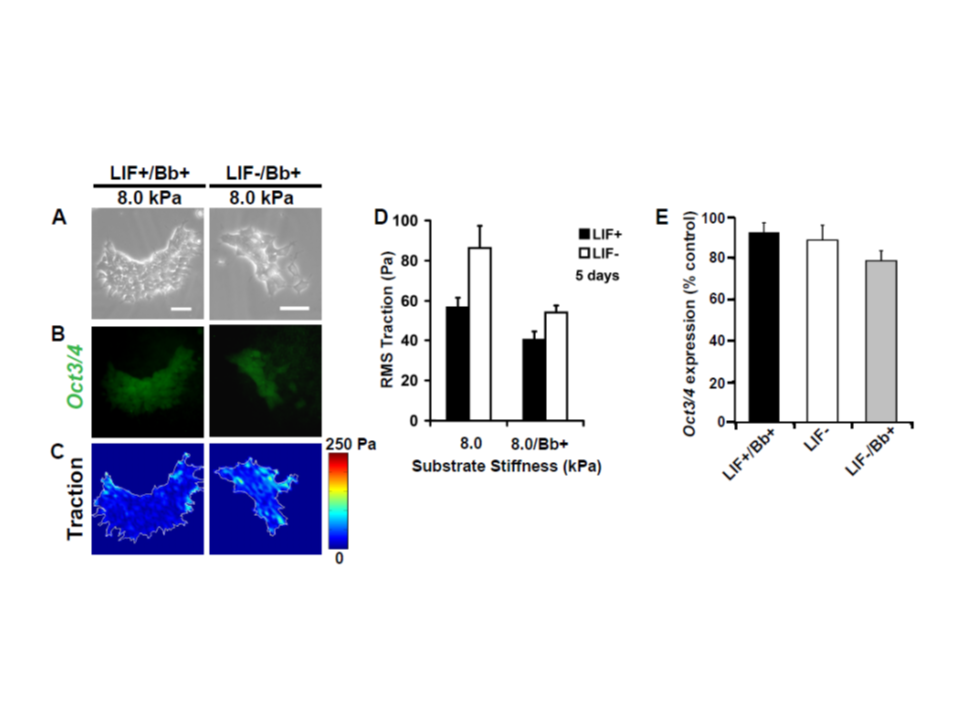

Supplement: Figure S5 — Blebbistatin (10 µM) treatment on 8 kPa substrates for 5 days decreases RMS tractions. (A–C) Blebbistatin treatment (Bb+) altered colony shape (A), Oct3/4 expression (B), and tractions (C). (D) For LIF+ conditions, adding blebbistatin downregulated tractions (p = 0.032; n = 10 colonies). Similary, for LIF− conditions, addition of blebbistatin decreased tractions (p = 0.03; n = 8 colonies). For convenience of comparison, data (without blebbistatin) are replots from part of Fig. 4E. Mean ± s.e.m. Bars, 50 µm. (E) Summarized data for Oct3/4 expression after blebbistatin treatment. Control: colonies on 8 kPa with LIF (n = 9). Blebbistatin significantly lowered the level of Oct3/4 expression in colonies without LIF (n = 6) when compared with the control (p<0.01). LIF withdrawal alone (n = 7) or blebbistatin added to LIF+ condition (n = 8) decreased Oct3/4 expression from the control only slightly but not significantly (p>0.25). Mean+/−s.e.m. (TIF) [file pone.0015655.s005.tif]
