# Supplementary material for: Soft Substrates Promote Homogeneous Self-Renewal of Embryonic Stem Cells via Downregulating Cell-Matrix Tractions
Source: PLoS One. 2010 Dec 13;5(12):e15655. doi: 10.1371/journal.pone.0015655 (PMC3001487; doi:10.1371/journal.pone.0015655)
Supplement: Text S1 — Additional Methods. (DOC) [file pone.0015655.s006.doc]

Supporting Information

Additional Methods

Primers for Gene Expression Analysis

Primer pairs used in this study have been described earlier: *Eras* [1], *Oct3/4* [2], *Esg1* and *Ef1α* [3], *Tcf15* and *Twist2* [4], *T* [5], *Sox2* [6], *Nanog* [7].

AP Staining

Mouse ESCs were fixed in Dent’s fixative (DMSO:100%Methanol=1:4) at -20 °C for 1hr, and washed with PBS for three times. The Alkaline Phosphate (AP) kit (Sigma-Aldrich 85L3R) was used according to the manufacturer’s instructions.

Immunofluorescent microscopy

Immunofluorescent microscopy was carried out essentially as described previously [8]. Briefly, mESCs cultured on glass-bottom culture dishes (MatTek corporation) were fixed with Dent’s fixative at -20 °C overnight, washed with PBST (0.1% Tween in PBS) twice and blocked with PBSMT (2% skimmed milk in PBST) for 1hr. Fixed mESCs were incubated with goat anti-mouse Nanog polyclonal antibody (R&D systems) diluted with PBMST at the 1:200 ratio at 4 °C overnight. Normal lamb serum was diluted with PBSMT at 1:1,000 and used as a negative control. After two washes with PBSMT for 30min each at 4 °C followed by three more washes at room temperature, Alexa Fluor 546 rabbit anti-goat IgG (Molecular Probes) was diluted with PBSMT at the 1:400 ratio and used as a secondary antibody. After five washes with PBSMT as before, stained mESCs were incubated with DAPI (5 µg/ml, Sigma) at room temperature for 15min, and mounted into glycerol gelatin (Sigma) for fluorescence microscopy (Leica DMI4000B).

Immunofluorescent microscopy for paraffin-embedded sections of teratomas was carried out as described already [9] with following modifications. Deparaffinized 4-6 µm sections were rehydrated with Histo-clear II (Fisher)-alcohol series, immersed in 10 mM sodium citrate buffer (pH 6.0) [10], and heated by a microwave oven (LG, 1200W) for 5 min at level 5, three times. Then, blocked sections were incubated at RT for 1.5 hr with culture supernatant from mouse anti-nestin monoclonal antibody (Rat-401 raised by Dr. Susan Hockfield), or goat anti-α-fetoprotein (Santa Cruz Biotechnology Inc.) or rabbit anti-α-smooth muscle actin polyclonal antibodies (Abcam) diluted with 2 % skimmed milk in PBS with 0.1 % Tween 20 (PBSMT) at 1:100. Normal mouse, rabbit, or lamb sera diluted with PBSMT at 1:250 were used as negative controls. After 3 washes with PBSMT at RT for 10 min each, sections were incubated at RT for 1.5 hr with Alexa Fluor 488-conjugated goat anti-mouse IgG (whole molecule; Invitrogen), or Alexa Fluor 546-conjugated rabbit anti-goat or goat anti-rabbit IgG (whole molecule; Invitrogen) diluted with PBSMT at 1:400. After 4 washes with PBSMT, sections were stained with 0.5mg/ml DAPI (Sigma) in PBS, and mounted with glycerol-gelatin (Sigma).

Serial Passaging on soft substrates

An undifferentiated mESC line, W4 (129/SvEv), was serially passaged on rigid dish and soft gel (0.6 kPa) under LIF +/- condition for over three months. Both rigid dish and soft gel were coated with type I collagen. W4 cells were thawed on mitotically inactivated primary mouse embryonic fibroblast. After recovery, W4 cells were passaged onto 0.1% gelatin coated polystyrene tissue culture dishes couple of times to remove feeders. Then W4 cells were plated on rigid dishes or soft gels. On soft gels, cells were initially plated at a high concentration which was found to be useful particularly for LIF- condition. On the very first passage, cells were first plated with LIF+ medium. LIF+ medium was withdrawn the following day from one of the soft gels and labeled as LIF- condition. From this point onward exogenous LIF was never added for the LIF- condition on the subsequent passages. W4 cells on gel were passaged 1:3 ratio on subsequent passages. The medium was changed every two days and passaged every 3-4 days.

References

1. Takahashi K, Mitsui K, Yamanaka S (2003) Role of ERas in promoting tumour-like properties in mouse embryonic stem cells. Nature 423: 541-545.
2. Nichols J, Zevnik B, Anastassiadis K, Niwa H, Klewe-Nebenius D et al. (1998) ****Formation of Pluripotent Stem Cells in the Mammalian Embryo Depends on the POU Transcription Factor Oct4.**** Cell 95: 379-91.
3. Tanaka TS, Kunath T, Kimber WL, Jaradat SA, Stagg CA et al. (2002) Gene expression profiling of embryo-derived stem cells reveals candidate genes associated with pluripotency and lineage specificity. Genome Res. 12: 1921-8.
4. Tanaka TS, Davey RE, Lan Q, Zandstra PW, Stanford WL (2008) Development of a gene-trap vector with a highly sensitive fluorescent protein reporter system for expression profiling. Genesis 46: 347-356.
5. Fujikura J, Yamato E, Yonemura S, Hosoda K, Masui S et al. (2002) Differentiation of embryonic stem cells is induced by GATA factors. Genes Dev. 16: 784-9.
6. Masui S, Nakatake Y, Toyooka Y, Shimosato D, Yagi R et al. (2007) Pluripotency governed by Sox2 via regulation of Oct3/4 expression in mouse embryonic stem cells. Nat. Cell Biol. 9: 625-635.
7. Mitsui K, Tokuzawa Y, Itoh H, Segawa K, Murakami M et al. (2003) The homeoprotein Nanog is required for maintenance of pluripotency in mouse epiblast and ES cells. Cell 113: 631-642.
8. Tanaka TS, Lopez de Silanes I, Sharova LV, Akutsu H, Yoshikawa T et al. (2006) Esg1, expressed exclusively in preimplantation embryos, germline, and embryonic stem cells, is a putative RNA-binding protein with broad RNA targets. Dev. Growth Differ. 48: 381–390.
9. Tanaka T. S., Lopez de Silanes I., Sharova L. V., Akutsu H., Yoshikawa T., Amano H., Yamanaka S., Gorospe M., and Ko M. S. H. (2006) Esg1, expressed exclusively in preimplantation embryos, germline, and embryonic stem cells, is a putative RNA-binding protein with broad RNA targets. Develop. Growth Differ. 48, 381-390.
10. Lan H. Y., Mu W., Nikolic-Paterson D. J., and Atkins R. C. (1995) A novel, simple, reliable, and sensitive method for multiple immunoenzyme staining: Use of microwave oven heating to block antibody crossreactivity and retrieve antigens. J. Histochem. Cytochem. 43, 97-102.
11. Engler A, Bacakova L, Newman C, Hategan A, Griffin M et al. (2004) Substrate compliance versus ligand density in cell on gel responses. Biophys J 86: 617-628.

Figure S1. Mouse ESCs were plated on collagen-1 (100 μg/ml) coated rigid dishes and cultured for 5 days in LIF+/- conditions. The colonies were immunostained for Oct3/4 and the alkaline phosphatase (AP) activity. Colonies exhibited similar phenotypes to the ones maintained on 40 μg/ml collagen-1.

Figure S2. Mouse embryonic stem cells (mESCs; OGR1) thawed and maintained on soft gels formed round and compact colonies as they did on feeders. Bright (*A*-*F*) and dark (*A*’-*F*’) field images are shown. (*A-A*’) ORG1 mESCs thawed on rigid dishes formed small spread colonies on day 3. However, *Oct3/4*::GFP expression at this stage were not significantly diminished. (*B*-*B*’) mESCs thawed on rigid dishes on day 6 showed appearance of spread and differentiated cells. The corresponding dark field image showed very low GFP expression. (*C*-*C*’) mESCs thawed on the soft gels started to form round and compact colony on day 3 with GFP expression. (*D*-*D*’) on day 6, these mESCs on the soft gel still formed very round and compact colonies with GFP uniformly expressed. (*E*-*E*’) On day 3, mESCs thawed on feeders appeared to have colonies of various shapes ranging from relatively round to somewhat flattened (white arrow in *E*). The flattened colony showed low GFP expression (arrow in *E*’). (*F*-*F*’) On day 6, mESCs formed relatively round colonies on feeders with GFP expression, except for the cells on the edge of the colony whose GFP expression was relatively low, showing early signs of differentiation. (*G*) Comparisons among the shapes of colonies on the rigid dish, the gel and the feeders by quantifying the colony shape factor [11]. The colony shape factor (=4πArea/Perimeter^2^; Area=colony projected area; Perimeter=perimeter length of a colony) measures to what extent the colony is similar to a true circle. A true circle has a value of unity. Data are mean ± s.e.m., *n*=29, 32, 30 colonies for the rigid dish, the gel and the feeders respectively. *p*<0.0001 between any two conditions. Bars, 100 µm.

Figure S3. Mouse ESCs maintained on soft gels under LIF+ and LIF- conditions formed a well-developed teratoma when transplanted into NOD-SCID mice subcutaneously. (*A*) Teratomas (dashed circles) are developed from mESCs cultured on the soft gel in the presence of LIF. (*B*) The teratoma on *left* is developed from mESCs on the soft gel, whereas the teratoma on *right* is from ones on rigid dishes in the absence of LIF. n=2 separate mice. The teratoma on right is significantly smaller in size. (*C*-*E*) Hematoxylin and Eosin (H & E) staining of sections from a teratoma of mESCs maintained on the soft gel with LIF shows the presence of cells from all three germ layers. Ne: Neural tissue (ectoderm); St: Striated muscle (mesoderm); Ci: Ciliated epithelium (endoderm).

Figure S4. Undifferentiated mouse ES cell line, W4 (129/SvEv), was serially passaged (images shown at passage 15) on rigid dishes and soft gels (0.6 kPa) under LIF +/- conditions for over three months. Even in the presence of LIF on rigid dishes, cells start to exhibit decreased *Oct3/4* expression and the AP activity accompanied by appearance of differentiated cells at the colony periphery (row 1). However, their self-renewal was maintained best on soft gels in the presence of LIF, evident by the high *Oct3/4* expression level, the high AP activity, and compact and round morphology (row 2). Remarkably, cells on soft gels also maintained self-renewal in the absence of LIF with sustained *Oct3/4* expression and the AP activity (row 3).

Figure S5. Blebbistatin (10 μM) treatment on 8 kPa substrates for 5 days decreases RMS tractions. (A-C) Blebbistatin treatment (Bb+) altered colony shape (A), Oct3/4 expression (B), and tractions (C). (D) For LIF+ conditions, adding blebbistatin downregulated tractions (*p*=0.032; n=10 colonies). Similary, for LIF- conditions, addition of blebbistatin decreased tractions (*p*=0.03; n= 8 colonies). For convenience of comparison, data (without blebbistatin) are replots from part of Fig. 4E. Mean ± s.e.m. Bars, 50 μm. (E) Summarized data for *Oct3/4* expression after blebbistatin treatment. Control: colonies on 8 kPa with LIF (n=9). Blebbistatin significantly lowered the level of *Oct3/4* expression in colonies without LIF (n=6) when compared with the control (p<0.01). LIF withdrawal alone (n=7) or blebbistatin added to LIF+ condition (n=8) decreased *Oct3/4* expression from the control only slightly but not significantly (p>0.25). Mean+/-s.e.m.
